# Indoor residual spraying practices against *Triatoma infestans* in the Bolivian Chaco: contributing factors to suboptimal insecticide delivery to treated households

**DOI:** 10.1186/s13071-021-04831-1

**Published:** 2021-06-16

**Authors:** Raquel Gonçalves, Rhiannon A. E. Logan, Hanafy M. Ismail, Mark J. I. Paine, Caryn Bern, Orin Courtenay

**Affiliations:** 1 Zeeman Institute and School of Life Sciences, University of Warwick, Coventry, CV4 7AL UK; 2 Liverpool School of Tropical Medicine, Department of Vector Biology, Faculty of Biological Sciences, Pembroke Place, Liverpool, L3 5QA UK; 3 Department of Epidemiology and Biostatistics, School of Medicine, University of California San Francisco, San Francisco, CA USA

**Keywords:** Chagas disease, *Triatoma infestans*, *Trypanosoma cruzi*, Vector control, Diagnostic, Indoor residual spraying, Insecticide quantification, Chaco, Bolivia

## Abstract

**Background:**

Indoor residual spraying (IRS) of insecticides is a key method to reduce vector transmission of *Trypanosoma cruzi*, causing Chagas disease in a large part of South America. However, the successes of IRS in the Gran Chaco region straddling Bolivia, Argentina, and Paraguay, have not equalled those in other Southern Cone countries.

**Aims:**

This study evaluated routine IRS practices and insecticide quality control in a typical endemic community in the Bolivian Chaco.

**Methods:**

Alpha-cypermethrin active ingredient (a.i.) captured onto filter papers fitted to sprayed wall surfaces, and in prepared spray tank solutions, were measured using an adapted Insecticide Quantification Kit (IQK™) validated against HPLC quantification methods. The data were analysed by mixed-effects negative binomial regression models to examine the delivered insecticide a.i. concentrations on filter papers in relation to the sprayed wall heights, spray coverage rates (surface area / spray time [m^2^/min]), and observed/expected spray rate ratios. Variations between health workers and householders’ compliance to empty houses for IRS delivery were also evaluated. Sedimentation rates of alpha-cypermethrin a.i. post-mixing of prepared spray tanks were quantified in the laboratory.

**Results:**

Substantial variations were observed in the alpha-cypermethrin a.i. concentrations delivered; only 10.4% (50/480) of filter papers and 8.8% (5/57) of houses received the target concentration of 50 mg ± 20% a.i./m^2^. The delivered concentrations were not related to those in the matched spray tank solutions. The sedimentation of alpha-cypermethrin a.i. in the surface solution of prepared spray tanks was rapid post-mixing, resulting in a linear 3.3% loss of a.i. content per minute and 49% loss after 15 min. Only 7.5% (6/80) of houses were sprayed at the WHO recommended rate of 19 m^2^/min (± 10%), whereas 77.5% (62/80) were sprayed at a lower than expected rate. The median a.i. concentration delivered to houses was not significantly associated with the observed spray coverage rate. Householder compliance did not significantly influence either the spray coverage rates or the median alpha-cypermethrin a.i. concentrations delivered to houses.

**Conclusions:**

Suboptimal delivery of IRS is partially attributable to the insecticide physical characteristics and the need for revision of insecticide delivery methods, which includes training of IRS teams and community education to encourage compliance. The IQK™ is a necessary field-friendly tool to improve IRS quality and to facilitate health worker training and decision-making by Chagas disease vector control managers.

**Graphic Abstract:**

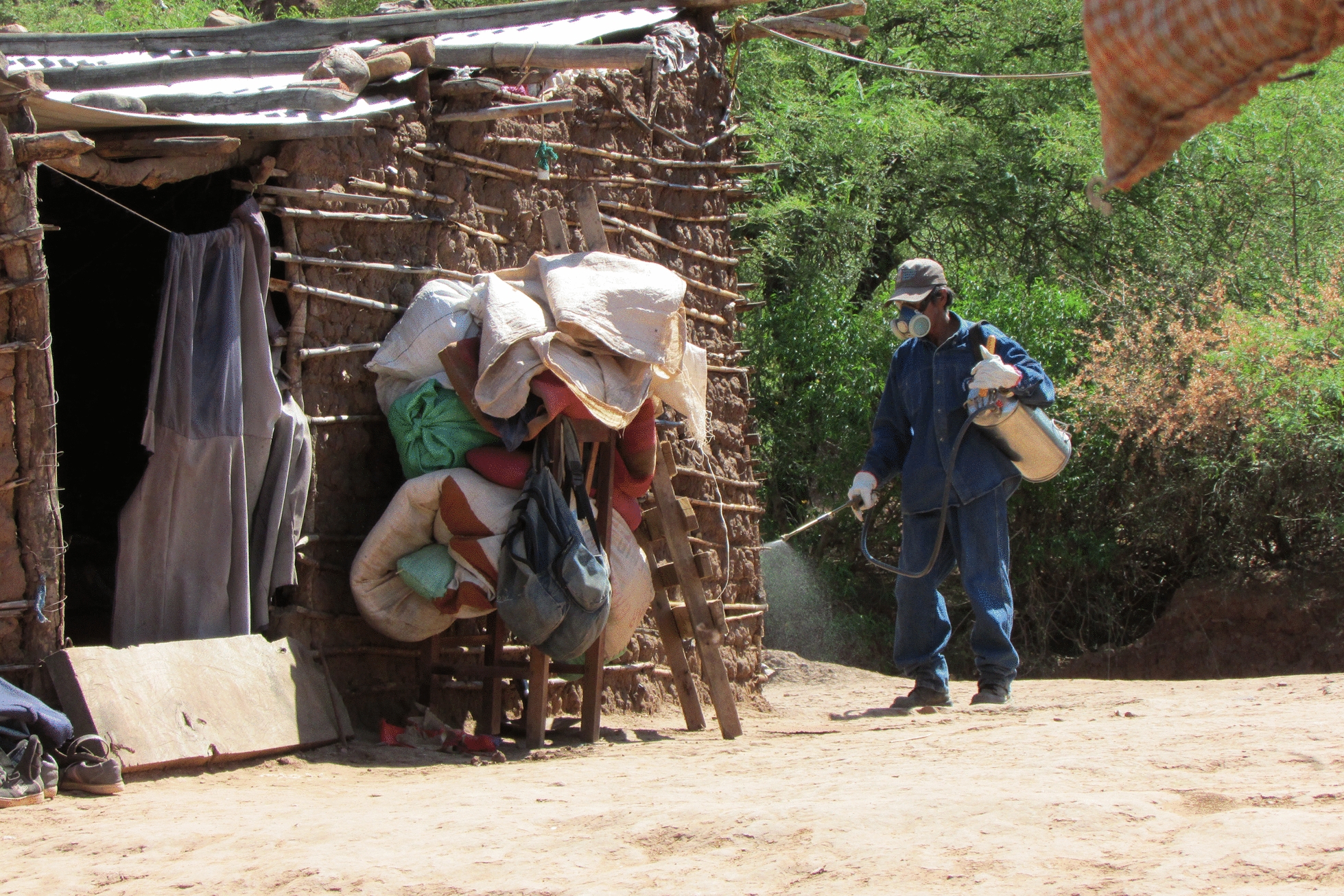

**Supplementary Information:**

The online version contains supplementary material available at 10.1186/s13071-021-04831-1.

## Introduction

Chagas disease results from infection with the parasite *Trypanosoma cruzi* (Kinetoplastida: Trypanosomatidae) which causes a range of pathologies in humans and other animals. In humans, acute symptomatic infection occurs within weeks to months of infection, characterised by fever, malaise, and hepatosplenomegaly. An estimated 20–30% of infections progress to chronic forms, most commonly cardiomyopathy, characterized by conduction system deficits, arrhythmias, left ventricular dysfunction, and eventually, congestive heart failure, and less frequently by gastrointestinal forms of the disease. These conditions develop over decades and are difficult to treat [1]. There is no vaccine.

The estimated global burden of Chagas disease in 2017 was 6.2 million, resulting in 7900 deaths and 232,000 all-age disability-adjusted life-years (DALYs) [2–4]. *T. cruzi* is transmitted by triatomine bugs (Hemiptera: Reduviidae) throughout Central and South America and parts of southern North America, which accounted for 30,000 (77%) of the total new cases in 2010 in Latin America [5]. Congenital transmission and infected blood transfusions are additional routes of infection occurring in non-endemic regions such as Europe and the USA. In Spain, for example, there are an estimated 67,500 infections among Latin American immigrants [6], at an annual cost to the healthcare system of $9.3 million USD [7]. In a Barcelona hospital, between 2004 and 2007, 3.4% of screened pregnant women immigrants from Latin American countries were seropositive for *T. cruzi* [8]. Thus, efforts to control vector transmission in endemic countries is fundamental to reducing the burden of disease also in countries without triatomine vectors [9]. Current control methods include indoor residual spraying (IRS) of insecticides to reduce domestic and peridomestic vector populations, maternal screening to detect and address congenital transmission, screening of donor blood banks and transplant organs, and education programmes [5, 10–12].

In the Southern Cone countries of South America, the main vector is *Triatoma infestans*. This species is predominantly endophilic and endophagic, with widespread breeding colonies inside households and animal sheds; household infestations are particularly abundant in poorly constructed buildings in which wall and ceiling crevices provide triatomine refuge [13, 14]. The Southern Cone Initiative (INCOSUR) has promoted a coordinated international effort to combat domestic infestation by *Tri. infestans* and other domiciled vectors using IRS [15, 16]. This has resulted in substantial reductions in Chagas disease incidence and consequent certification by WHO of interrupted vector transmission in some countries (Uruguay, Chile, some parts of Argentina, and Brazil) [10, 15].

Despite the successes of INCOSUR, vector transmission of *T. cruzi* persists in the American Gran Chaco, a seasonal dry forest ecosystem of 1.3 million km^2^ that straddles the borders of Bolivia, Argentina, and Paraguay [10]. The inhabitants in this region are some of the most marginalised, living in extreme poverty with little access to health care [17]. The incidence of *T. cruzi* infection and vector transmission amongst these communities is the highest in the world [5, 18–20], where 26–72% of houses are infested with *Tri. infestans* [13, 21], and 40–56% of *Tri. infestans* are infected with *T. cruzi* [22, 23]. The majority (> 93%) of all vector-transmitted Chagas disease cases in the Southern Cone region occur in Bolivia [5].

IRS is currently the only widely deployed method to reduce human–*Tri. infestans* contact, being a historically proven strategy to reduce the burden of some vector-borne human diseases [24, 25]. The proportion of houses in a community with *Tri. infestans* infestation (Infestation Index) is one key measure used by health authorities to guide decision-making about IRS deployment and, importantly, to justify the treatment of chronically infected children without the risk of reinfection [16, 26–29]. Factors affecting IRS effectiveness, and the persistence of vector transmission in the Grand Chaco region, are variously attributed to poor building construction [19, 21], suboptimal IRS implementation practices and infestation surveillance methods [30], low public compliance with IRS requirements [31], short residual activity of insecticide formulations [32, 33], and *Tri. infestans* resistance and/or reduced susceptibility to insecticides [22, 34].

Synthetic pyrethroid insecticides are commonly used for IRS, as they are lethal to susceptible triatomine populations. At low concentrations, pyrethroid insecticides are also used as an irritant to flush out the vectors from wall crevices for purpose of surveillance [35]. Quality control studies of IRS practices are limited, but elsewhere indicate substantial variance in insecticide active ingredient (a.i.) concentrations delivered to houses, with levels frequently below the effective target concentration range [33, 36–38]. One reason for the lack of quality control studies is that high-performance liquid chromatography (HLPC), the gold-standard method for measuring insecticide a.i. concentrations, is technically challenging, costly, and generally not suited to endemic community settings. Recent advances in laboratory assays now provide alternative and relatively cheap methods to assess insecticide delivery and IRS practices [39, 40].

This study aimed to measure the variation in insecticide concentrations during routine IRS campaigns against *Tri. infestans* in the Bolivian Chaco. Insecticide a.i. concentrations were measured in prepared formulations in spray tanks, and in filter paper samples collected from sprayed houses. Factors potentially affecting delivery of insecticides to houses were also evaluated. In so doing, we adapted a chemical colorimetric assay to quantify pyrethroid concentrations in these samples.

## Methods

### Study site

**Fig. 1:**
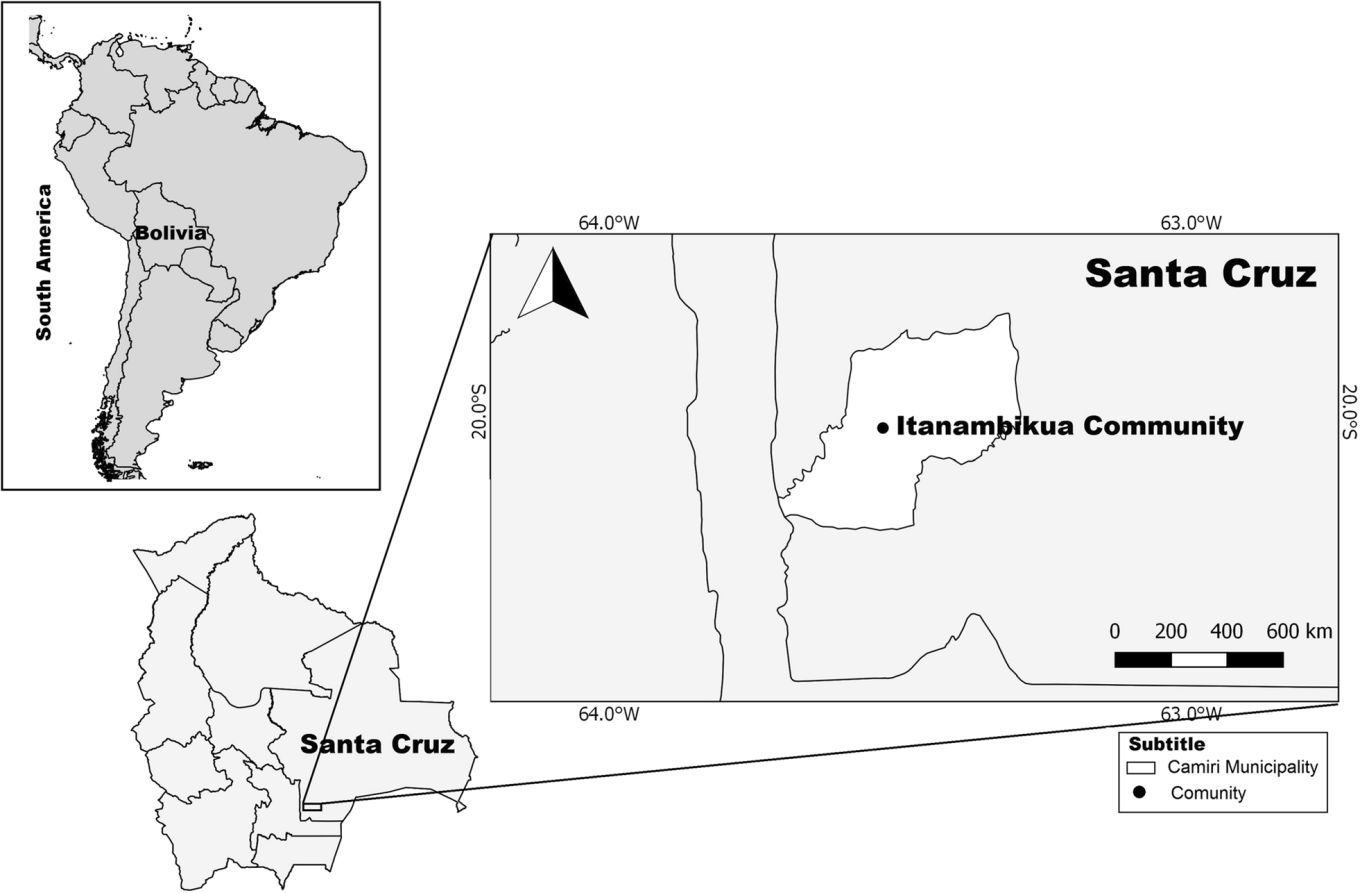
Location of the Itanambikua study site in Camiri Municipality, Santa Cruz Department, Bolivia

The study was conducted in Itanambikua (20º1′5.94″S; 63º30′41″W) located in Camiri Municipality, Santa Cruz Department, Bolivia (Fig. 1). The area forms part of the American Gran Chaco, characterised by seasonal dry forest, with temperature of 0–49 °C and rainfall of 500–1000 mm/year [41]. Itanambikua is one of 19 ethnic Guarani communities in the municipality, holding a population of approximately 1200 residents living in 220 houses which are constructed largely of sun-baked bricks (adobe), traditional wattle-and-daub (locally called *tabique*), wood, or a mixture of these materials. Additional buildings and structures near the houses include animal sheds, storehouses, kitchens, and latrines constructed of similar materials. The local economy is based on subsistence farming, mainly maize and peanuts, and small-scale breeding of poultry, pigs, goats, ducks, and fish, the household surplus of which is sold in the local commercial town of Camiri (about 12 km distance). Camiri town also provides some employment to the community primarily in the building industry and domestic services.

During the current study, the prevalence of *T. cruzi* infection in children (2–15 years old) in Itanambikua was 20% [20]. This was similar to the childhood infection seroprevalence reported for nearby Guarani communities, also showing an increase in prevalence with age, with the vast majority of residents > 30 years old infected [19]. Vector transmission is considered the main route of infection in these communities, the predominant vector being *Tri. infestans* which colonises houses and outbuildings [21, 22].

Records of IRS campaigns conducted in Itanambikua prior to this study were not available from the recently elected municipality health authorities; however, reports from nearby communities clearly indicated that IRS campaigns were sporadic in this municipality, starting in 2000 with blanket spraying using 20% alpha-cypermethrin in 2000 and 2003, followed by focal spraying of infested houses from 2005 to 2009 [22] and non-systematic spraying between 2009 and 2011 [19].

## Study design

### IRS practices

In this community, IRS was performed by three resident trained health workers using alpha-cypermethrin suspension concentrate [SC] 20% formulation (Alphamost^®^, Hockley International Ltd., Manchester, UK). The insecticide was prepared at a delivery target concentration of 50 mg a.i./m^2^ following the requirements of the Chagas Disease Control Programme, Santa Cruz Administrative Department (*Servicio Departamental de Salud*—SEDES). The insecticide was applied using a Guarany^®^ knapsack sprayer tank (Guarany Indústria e Comércio Ltda, Itu, São Paulo, Brazil) with 8.5L useful capacity (tank code: 0441.20), equipped with a flat fan nozzle with a nominal flow rate of 757 ml/min, producing a spray at an angle of 80° at a standard tank pressure of 280 kPa. The same health workers that mixed the spray tanks also sprayed the houses. These workers had previously received training from the local municipality health authorities in insecticide preparation and insecticide delivery to spray both the inside and outside walls of houses. They were also advised to request that householders empty their houses of all belongings including furniture (except for bed frames) at least 24 h before IRS was scheduled, the aim being to permit full access to the house interior for spraying. Compliance with this request was measured as outlined below. Householders were also advised to wait until the sprayed walls appeared dry before re-entering their house, as recommended [42].

### Insecticide concentrations delivered to houses

To quantify the alpha-cypermethrin a.i. concentrations delivered to houses, researchers fitted filter papers (Whatman no. 1; 55 mm diameter) to the wall surfaces of 57 houses just prior to IRS. All houses that had received IRS at the time were recruited (25/25 houses in November 2016 and 32/32 houses in January–February 2017). These included 52 adobe houses and 5 *tabique* houses. Eight to nine filter papers were fitted to each house divided between three wall heights (0.2, 1.2, and 2 m above ground level), on each of three walls selected in an anticlockwise direction starting from the main door. This provided three replicates at each wall height as recommended to monitor insecticide a.i. delivery [43]. Immediately after insecticide application, filter papers were collected by researchers and left to dry protected from direct sunlight. Once dry, filter papers were wrapped in Sellotape to protect and maintain the insecticide on the covered surface and then wrapped in aluminium foil for storage at 7 °C until testing. Of the total 513 filter papers collected, 480 from 57 houses were available for testing, i.e. 8–9 filter papers per house. The tested samples included 437 filter papers from 52 adobe houses, and 43 filter papers from 5 *tabique* houses. This sample was in proportion to the relative abundance of house construction types in the community (76.2% [138/181] adobe and 11.6% [21/181] *tabique*) as recorded by house-to-house survey as part of this study. The Insecticide Quantification Kit (IQK™) assay adapted for filter papers, and its validation against HPLC, is described in the Additional file 1. The target insecticide concentration was 50 mg a.i./m^2^, allowing for ± 20% tolerance (i.e. 40–60 mg a.i./m^2^).

### Insecticide concentrations in the Guarany^®^ spray tank

Concentrations of a.i. were quantified in 29 spray tanks prepared by the health workers. We sampled 1–4 prepared tanks per day, with a mean of 1.5 (range: 1–4) tanks prepared per day over an 18-day period; the sampling order followed the day-to-day progression of the health workers during November 2016 and January–February 2017. Immediately after rigorous mixing of the formulation, 2 ml of the solution was collected from the surface content. The 2 ml sample was then vortexed for 5 min in the laboratory, and two 5.2 μl sub-samples collected and tested using the IQK™ as described (see Additional file 1).

The sedimentation rates of the insecticide a.i. were measured in four spray tanks purposefully selected to represent higher, lower, and within target range initial (time zero) a.i. concentrations. Three sub-samples of 5.2 μl were collected from the surface layer of each vortexed 2 ml sample at intervals of 1 min for 15 consecutive minutes post-mixing. The target tank solution concentration was 1.2 mg a.i./ml ± 20% (i.e. 0.96–1.44 mg a.i./ml) which was equivalent to achieve the target concentration delivery to filter papers as described above.

### Assessment of spraying practices

To understand the relationship between insecticide spraying activities and insecticide delivery, one researcher (RG) accompanied two of the community IRS health workers during the routine IRS deployment to 87 houses (the 57 recruited houses described above, in addition to 30 of 43 houses sprayed in March 2016). Thirteen of these 43 houses were excluded from analyses: six house owners declined, and seven houses were only partially sprayed. Details of the total surface area (m^2^) inside and outside of houses to spray were measured, and the total time (minutes) that the health worker spent spraying was cryptically recorded. These raw data were used to calculate the spray rate defined as unit surface area sprayed per minute (m^2^/min). From these data, the observed/expected spray coverage rate ratio was also calculated as a relative measure where the recommended expected spray rate is 19 m^2^/min ± 10% [44] for the spray equipment specifications. For the observed/expected ratio, the tolerance range was 1 ± 10% (0.8–1.2).

Filter papers were fitted to the walls of the 57 houses as described. To test whether the visual presence of filter papers influenced the health worker’s spraying rates, spray rates in these 57 houses were compared to spray rates in the 30 houses treated in March 2016 which did not have filter papers fitted. Insecticide concentrations were only measured in houses fitted with filter papers.

### Householder compliance

Householders’ compliance with the request to empty their houses prior to IRS was recorded for 55 houses, which included the 30 houses sprayed in March 2016 and the 25 houses sprayed in November 2016. Their level of compliance was measured on a semi-quantitative scale of 0–2 (0 = all or the majority of contents were left in the house; 1 = most contents were removed; 2 = houses were completely emptied). The influence of owner compliance on spray rates and insecticide a.i. concentrations was examined.

### Statistical power calculations

The statistical power was calculated to detect significant deviations from expected alpha-cypermethrin a.i. concentrations delivered to filter papers and to detect significant differences in insecticide concentrations and spray rates between categorised paired house groups. The minimum statistical power (for *α* = 0.05) was calculated for the smallest numbers of recruited houses for any classification group identified at baseline (i.e. fixed sample sizes). Thus, one-sample comparison of mean insecticide concentrations in 17 recruited houses (classified as owner non-compliant) had a power of 98.5% to detect a 20% deviation from the expected mean target concentration of 50 mg a.i./m^2^, where the variance (SD = 10) was inflated based on published observations elsewhere [37, 38]. The equivalent power to compare insecticide concentrations in spray tanks matched to houses (*n* = 21) was > 90%.

Two-sample comparisons of mean insecticide concentrations delivered to *n* = 10 and *n* = 12 houses, or mean spray rates in *n* = 12 and *n* = 23 houses, gave a statistical power of 66.2% and 86.2% to detect a 20% difference from the expected values of 50 mg a.i./m^2^ and 19 m^2^/min, respectively. Potentially large variance values in spray rates (SD = 3.5) and in insecticide a.i. concentrations (SD = 10) in each group were conservatively assumed. For equivalent comparisons of spray rates in houses with (*n* = 57) and without (*n* = 30) fitted filter papers, the statistical power was > 90%. All power calculations were performed using the SAMPSI routine in STATA v15.0 software [45]).

### Statistical analyses

Alpha-cypermethrin a.i. concentrations on filter papers collected from houses were examined by fitting the data to mixed-effects multivariate negative binomial models (MENBREG routine in STATA v.15.0) with wall position (three levels) nested within houses as random effects. These models were used to test for variations associated with sprayed wall height (three levels); spray rate (m^2^/min), date of IRS application, and the health worker’s identity (two levels). Generalised linear models (GLMs) were used to test the association between the median filter paper alpha-cypermethrin a.i. concentration delivered per house and in the associated spray tank solution. Sedimentation of the insecticide a.i. concentrations in the spray tank solution over time were similarly examined including the starting value (at time zero) as the model offset, testing the tank ID × time (days) interaction term. Outlier data points *x* were identified by applying the standard Tukey fence boundary rule where *x* < Q_1_ − 1.5 × IQR or *x* > Q3 + 1.5 × IQR. Spray rate values for seven houses and the insecticide a.i. median concentration value for one house were excluded from statistical analyses as indicated.

## Results

### Validation of the IQK™ test

**Fig. 2:**
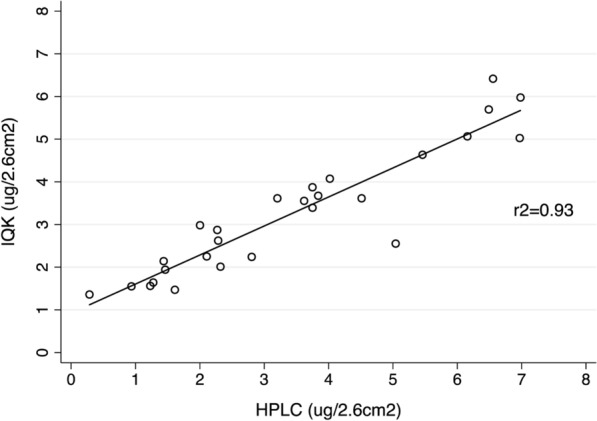
The association of the alpha-cypermethrin a.i. concentrations on filter paper samples collected from houses post-IRS, quantified by both HPLC and by the IQK™ (*n* = 27 filter papers from three houses)

The accuracy of IQK™ chemistry to quantify alpha-cypermethrin a.i. concentrations was validated by comparing the values of 27 filter paper samples from three houses tested by both IQK™ and HPLC (the gold standard), which showed a strong correlation (*r* = 0.93; *p* < 0.001) (Fig. 2).

### Alpha-cypermethrin concentrations delivered to houses

**Fig. 3:**
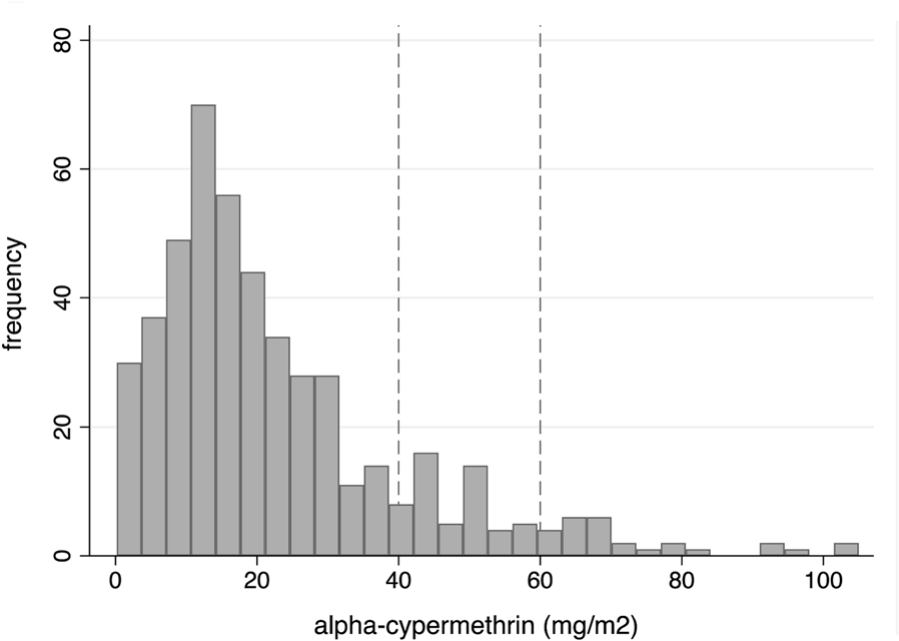
Frequency distribution of alpha-cypermethrin a.i. concentrations on filter papers collected from IRS-treated houses (*n* = 57 houses). The vertical lines represent the alpha-cypermethrin a.i. concentration target range (50 mg ± 20% a.i./m^2^)

**Fig. 4:**
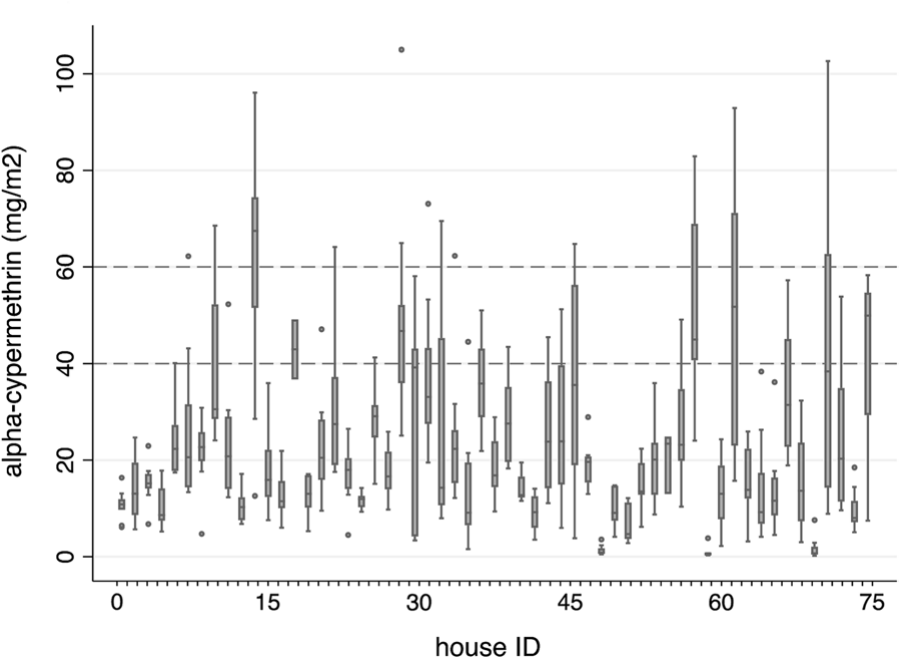
Median concentrations of alpha-cypermethrin a.i. on 8–9 filter papers per house collected from IRS-treated houses (*n* = 57 houses). The horizontal lines represent the alpha-cypermethrin a.i. concentration target range (50 mg ± 20% a.i./m^2^). Error bars represents the lower and upper median adjacent values

A total 480 filter papers collected from 57 houses were tested by the IQK™. On filter papers, alpha-cypermethrin quantities ranged from 0.19 to 105.0 mg a.i./m^2^ (median 17.6, IQR: 11.06–29.78). Of these, only 10.4% (50/480) were within the target concentration of 40–60 mg a.i./m^2^ (Fig. 3). The majority of the samples, 84.0% (403/480), were < 40 mg a.i./m^2^, and 5.6% (27/480) were > 60 mg a.i./m^2^. The median concentrations per house calculated for the 8–9 tested filter papers collected per house varied by an order of magnitude, with a median of 19.6 mg a.i./m^2^ (IQR: 11.76–28.32, range: 0.60–67.45). Only 8.8% (5/57) of houses received the expected insecticide concentration; 89.5% (51/57) were lower, and 1.8% (1/57) were higher, than the target range limits (Fig. 4).

The median concentrations delivered to filter papers at wall heights of 0.2, 1.2 and 2.0 m were 17.7 mg a.i./m^2^ (IQR: 10.70–34.26), 17.3 mg a.i./m^2^ (IQR: 11.43–26.91), and 17.6 mg a.i./m^2^ (IQR: 10.85–31.37), respectively (illustrated in Additional file 2). Controlling for the IRS date, the mixed-effects model did not indicate significantly different concentrations between wall heights (*z* < 1.83, *p* > 0.067) or significant modification by spray date (*z* = 1.84 *p* = 0.070). The median concentrations delivered to the five *tabique* houses were not dissimilar to those delivered to the 52 adobe houses (*z* = 0.13; *p* = 0.89).

### Alpha-cypermethrin concentrations in spray tank preparations

**Fig. 5:**
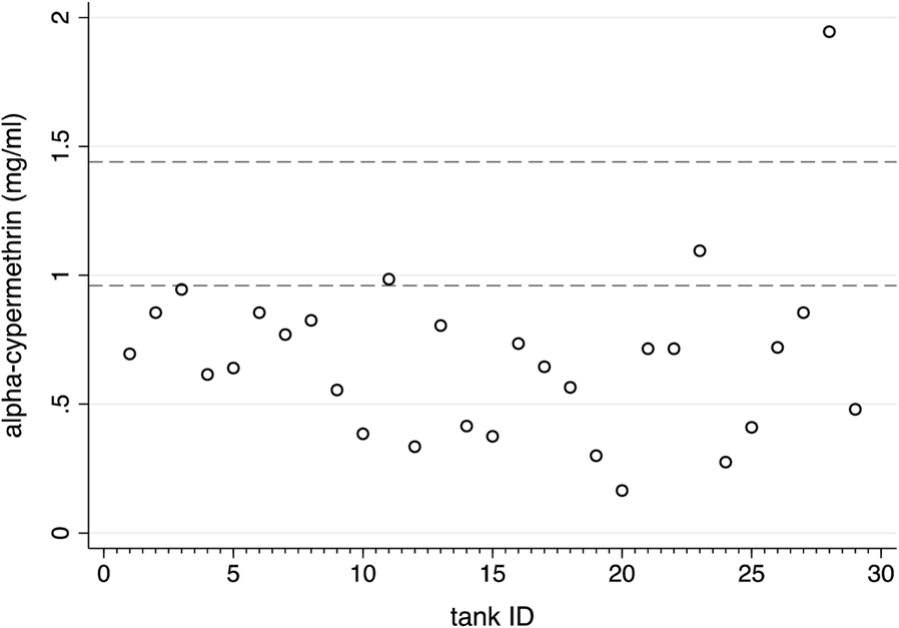
Median concentrations of alpha-cypermethrin a.i. measured in each of 29 spray tanks preparations. Horizontal lines indicate the recommended a.i. concentrations for spray tanks (0.96–1.44 mg/ml) to achieve a target a.i. concentration range in houses of 40–60 mg/m^2^

The a.i. concentrations in 29 independently prepared Guarany^®^ spray tanks, sampled just prior to IRS application, varied by a magnitude of 12.1, from 0.16 mg a.i./ml to 1.9 mg a.i./ml per tank (Fig. 5). Only 6.9% (2/29) of spray tanks contained a.i. concentrations within the target dose range of 0.96–1.44 mg a.i./ml, and 3.5% (1/29) of the tanks were > 1.44 mg a.i./ml.

**Fig. 6:**
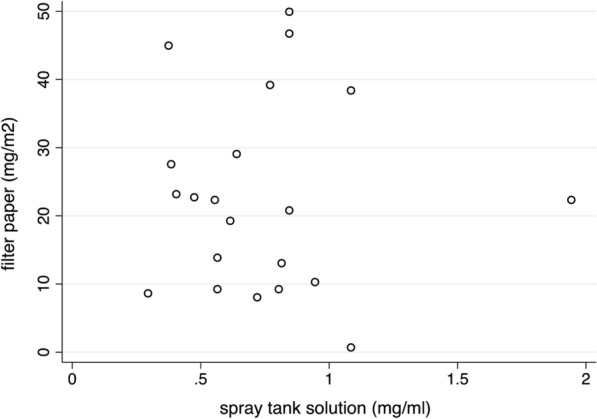
The association between the concentration of alpha-cypermethrin a.i. on 8–9 filter papers collected from IRS-treated houses, and a.i. concentrations in the independently prepared spray tank solution used to treat each of the houses (*n* = 21)

Of these 29 examined spray tanks, 21 were matched to spray 21 houses. The median a.i. concentrations delivered to houses was not associated with the concentrations in the individual spray tanks used to treat the house (*z* = −0.94, *p* = 0.345), reflected in a low correlation (*r*_Sp_^2^ = −0.02) (Fig. 6).

### Sedimentation of the alpha-cypermethrin a.i. in spray tank preparations

**Fig. 7:**
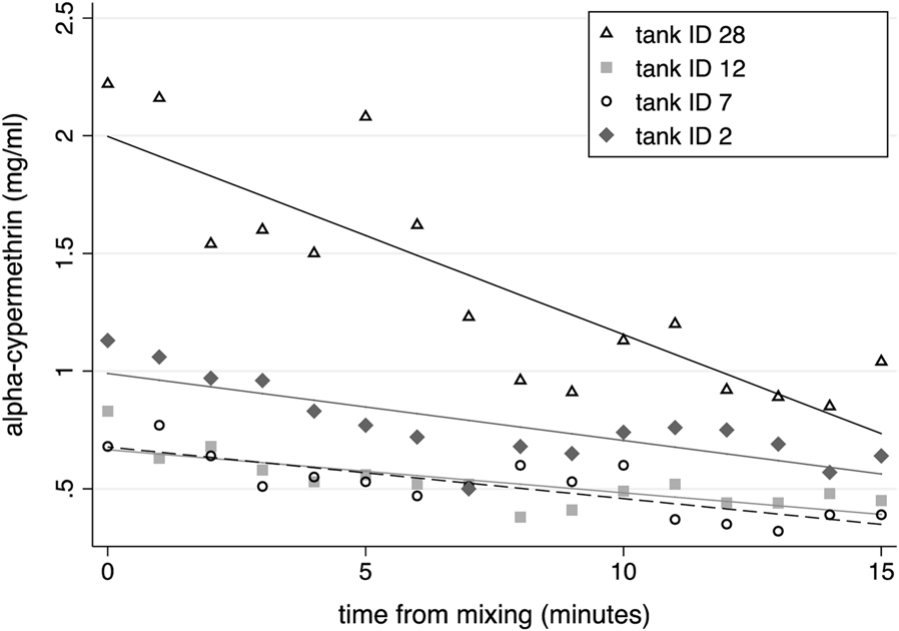
Sedimentation rates of alpha-cypermethrin a.i. in four spray tanks measured at 1-min intervals for 15 min following rigorous mixing of the tank solution. The lines representing the best fit to the data are shown for each tank. Observed values (points) represent the median of three sub-samples

The a.i. concentrations in the surface solution of four spray tanks sampled immediately after vortexing (at time 0) varied by a magnitude of 3.3 (0.68–2.22 mg a.i./ml) (Fig. 7). These values were within the target range for one tank, above target for one, and below target for the other two tanks. Thereafter, the insecticide a.i. concentrations in all four tanks significantly declined over the subsequent 15 min follow-up sampling (*b* = −0.018 to −0.084; *z* > 5.58; *p* < 0.001). Accounting for the individual tank starting values, the tank ID x time (minutes) interaction term was not significant (*z* = −1.52; *p* = 0.127). Across the four tanks, the mean loss in insecticide mg a.i./ml was 3.3% (95% CL 5.25, 1.71) per minute, amounting to 49.0% (95% CL 25.69, 78.68) after 15 min (Fig. 7).

### Variation in spray coverage rates

**Fig. 8:**
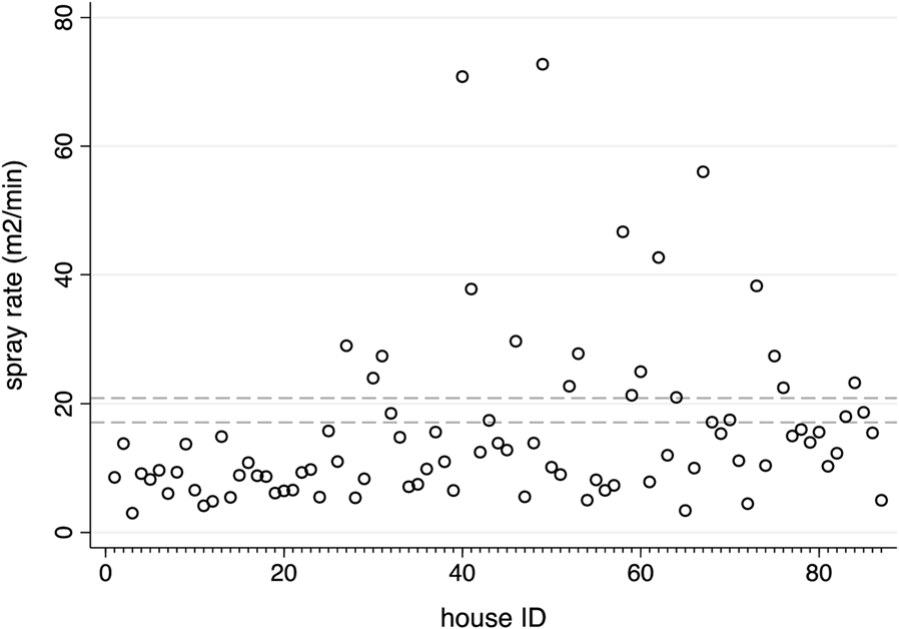
Observed spray rates (min/m^2^) in IRS-treated houses (*n* = 87). Reference lines indicate the expected 19 m^2^/min (± 10%) spray rate tolerance range recommended for the spray tank equipment specifications

The wall surface area for potential IRS treatment was a median 128 m^2^ (IQR: 99.0–210.0, range: 49.1–480.0) per house, and health workers spent a median 12 min (IQR: 8.2–17.5, range: 1.5–36.6) spraying each house (*n* = 87). The observed spray coverage rates ranged between 3.0 and 72.7 m^2^/min (median: 11.1; IQR: 7.90–18.00) in these houses (Fig. 8). Excluding outliers, the spray rates were compared to the WHO recommended spray rate of 19 m^2^/mi* n* ± 10% tolerance range (17.1–20.9 m^2^/min). Only 7.5% (6/80) of houses were within this range; 77.5% (62/80) were lower, and 15.0% (12/80) were greater. No association was detected between the median a.i. concentrations delivered to houses and the observed spray coverage rate (*z* = −1.59, *p* = 0.111, *n* = 52 houses).

The observed/expected spray coverage rate ratio was outside of the 1 ± 10% tolerance range for 80% of the 80 houses, with 71.3% (57/80) of houses lower and 11.3% (9/80) higher, and 16 houses falling within the tolerance range. The frequency distribution of the observed/expected ratio values is shown in Additional file 3.

Significant variation was detected in the median spray rates of the two health workers that routinely performed IRS, 9.7 m^2^/min (IQR: 6.58–14.85, *n* = 68) versus 15.5 m^2^/min (IQR: 13.07–21.17, *n* = 12) (*z* = 2.45, *p* = 0.014, *n* = 80) (illustrated in Additional file 4A), and also in their observed/expected spraying rate ratios (*z* = 2.58, *p* = 0.010) (illustrated in Additional file 4B).

### Presence/absence of filter papers

Excluding outliers, only one health worker sprayed the 54 houses with filter papers fitted. The median spray coverage rate in these houses was 9.23 m^2^/min (IQR: 6.57–13.80) compared to 15.4 m^2^/min (IQR: 10.40–18.67) in the 26 houses without fitted filter papers (*z* = −2.38, *p* = 0.017).

### Householder compliance

Householder compliance with the request to empty their house to allow access for IRS delivery varied: 30.9% (17/55) did not empty the house; 41.8% (23/55) semi-emptied the house, and 27.3% (15/55) fully emptied the house.

The observed spray coverage rates of non-emptied houses (17.5 m^2^/min, IQR: 11.00–22.50) tended to be higher than those for both semi-emptied houses (14.8 m^2^/min, IQR: 10.29–18.00) and fully emptied houses (11.7 m^2^/min, IQR: 7.86–15.36), though the difference was not significant (*z* > −1.58; *p* > 0.114, *n* = 48) (illustrated in Additional file 5A). Similar results were obtained when accounting for the variation associated with the presence or absence of filter papers which proved not to be a significant covariate in this model.

The absolute time spent spraying houses in these three groups did not differ among houses (*z* < −1.90, *p* > 0.057), whereas the median surface areas were different: fully emptied houses (104 m^2^ [IQR: 60.0–169.0 m^2^]) were statistically smaller than non-emptied houses (224 m^2^ [IQR: 174.0–284.0 m^2^] and semi-emptied houses (132 m^2^ [IQR: 108.0–384.0 m^2^] (*z* > 2.17; *p* < 0.031, *n* = 48). The average fully-emptied house was about half the size (surface area) of a non-emptied or semi-emptied house.

For the relatively small numbers of houses (*n* = 25) for which there were both compliance and insecticide a.i. data, no differences were detected in the median a.i. concentrations delivered to houses between these compliance categories (*z* < 0.93, *p* > 0.351), as illustrated in Additional file 5B. Similar results were obtained when accounting for the presence/absence of filter papers and the observed spray coverage rates (*n* = 22).

## Discussion

This study evaluated IRS practices and procedures in a typical rural community in the Bolivian Gran Chaco, where chronic vector-borne transmission persists [20]. The alpha-cypermethrin a.i. concentrations delivered during routine IRS varied substantially between houses, individual filter papers within houses, and between independent spray tanks prepared to achieve the same delivery concentration of 50 mg a.i./m^2^. Only 8.8% of houses (10.4% of filter papers) received concentrations within the target range of 40–60 mg a.i./m^2^, the majority (89.5% and 84% respectively) being below the lower tolerable limits.

A potential contributing factor to the suboptimal delivery of alpha-cypermethrin to houses is inaccuracies in the insecticide dilution and level of inconsistency of suspensions prepared in spray tanks [38, 46]. In the current study, observation of the health workers by the researchers confirmed that they followed the insecticide preparation formulas and training by SEDES to vigorously mix the solutions once diluted in the spray tank. Nonetheless, analysis of the tanks’ contents demonstrated a.i. concentrations that varied 12-fold; only 6.9% (2/29) of the tank solutions tested were within the target range. To investigate further, the spray tank surface solutions were quantified under laboratory conditions. This showed a linear decline of 3.3% in alpha-cypermethrin a.i. per minute after mixing, accumulating in a 49% (95% CL 25.7, 78.7) loss of a.i. after 15 min. High sedimentation rates due to aggregation of insecticide suspension formed from the dilution of wettable powder (WP) formulations is not uncommon (e.g. for DDT [37, 47]), and the current study further indicates this issue for a pyrethroid SC formulation. Suspension concentrates are widely used for IRS, and as for all insecticide formulations, their physical stability depends on many factors, particularly the a.i. compound particle size and other ingredients. Sedimentation can be affected also by the total hardness of water used to prepare the suspension, a factor that is difficult to control under field conditions. In this study site, for example, water access is restricted to the local river, which experiences seasonal fluctuations in flow rates and soil particle suspension. Methods to control the physical stability of SC formulations are under investigation [48]. Notwithstanding, SC formulations are successfully deployed to reduce domestic infestations of *Tri. infestans* in other regions of Latin America [49].

Inappropriate preparation of insecticide formulations has been reported in other vector control programmes. For example, in the Indian visceral leishmaniasis control programme, of 51 monitored spray teams, only 29% prepared and mixed DDT solutions correctly, and none filled the spray tanks according to guidelines [50]. Assessment in Bangladesh villages showed similar trends: only 42–43% of sub-district IRS teams prepared the insecticide and filled the spray tanks according to protocol; in one sub-district, this value was only 7.7% [46].

The observed variation in a.i. concentrations delivered to houses is also not unique. In India, only 7.3% (41/560) of sprayed houses received DDT at target concentrations, with similarly large variations within and between houses [37]. In Nepal, the average filter paper received 1.74 mg a.i./m^2^ (range: 0.0–17.5 mg/m^2^) which was only 7% of the target concentration (25 mg a.i./m^2^) [38]. HPLC analysis of filter papers revealed extensive variations in deltamethrin a.i. of 12.8–51.2 mg a.i./m^2^ delivered to walls, and 4.6–61.0 mg a.i./m^2^ delivered to roofs of Paraguayan Chaco houses [33]. In Tupiza, Bolivia, the Chagas control programme reported deltamethrin concentrations of 0.0–59.6 mg/m^2^ delivered to five houses, quantified by HPLC [36].

In contrast, IRS campaigns elsewhere report within target concentrations. For example, in Bioko Island, Republic of Equatorial Guinea, HPLC analysis of pirimiphos-methyl a.i. collected onto glue dots fitted to house walls demonstrated that 82% (49/60) of houses received the recommended concentrations of 5 g/m^2^ [51]. And in Vanuatu Island, South West Pacific, 83% (27/30) of houses sprayed with lambda-cyhalothrin received the expected concentrations as evaluated by the IQK™ [39].

The volume of insecticide released from the spray tank is dependent of the spray nozzle specifications, the pressure inside the tank, and time spent spraying the target surface [44]. The WHO recommended spray coverage rate that health workers should achieve using a flat spray nozzle (discharging 757 ml/minute at standard tank pressure of 280 kPa), is approximately 19 m^2^/min [44]. The spray coverage rates cryptically recorded during the current study indicated that only 7.5% (6/80) of houses fell within the expected tolerable range of 17.1 to 20.9 m^2^/min (i.e. 19 m^2^/min ± 10%). However, the observed spray coverage rates were not significantly associated with the median a.i. concentrations delivered to filter papers in houses, despite some variation in spraying practice between the two health workers monitored during this study.

The observed spray coverage rates in 77.5% (62/80) of houses were lower than the recommended expected value of 19 m^2^/min which further identifies likely gaps in health workers’ training in the IRS procedures. Hypothetically, the lower than expected spray coverage rates could be interpreted as potential delivery of more, rather than less, insecticide per unit surface area per unit time, assuming a constant rate of discharge. If this was the case, then it may have unintentionally lessened the disparity between the delivered and target a.i. concentrations in this study. In this case, the high sedimentation rates observed in prepared spray tanks was the dominant factor in the suboptimal delivery of insecticide.

The locally recruited health workers (residents of the Guarani communities) in this study received a single-day training course by SEDES, including basic instructions in insecticide preparation and delivery using a Guarany^®^ sprayer, and were provided with the insecticide. The authors did not identify any training manual, local or national IRS guidelines, or community records of annual IRS coverage. As part of the training, health workers were advised to request that householders empty their houses of all belongings, including heavy furniture, in order to facilitate access for IRS delivery. Compliance with this request is reported to affect the quality and insecticide coverage in houses [52]. In this study, compliance was variable; only 27.3% of the residents fully complied, whereas the remainder of houses were either not emptied or only partially emptied. Differences in spray coverage rates were not significantly associated with compliance; however, houses that were fully emptied were approximately half to three quarters the size of houses in the other categories, and hence greater compliance tended to be amongst the residents living in smaller houses, presumably because smaller houses are easier to empty. Such distinctions between house sizes and levels of compliance may prove immaterial, since in our relatively small sample we did not detect statistical differences in the delivered alpha-cypermethrin a.i. concentrations between these compliance categories, though the sample was limited. Further studies are warranted. We also acknowledge that we did not discount the estimated total surface area of houses by specific obstacles (e.g. furniture) not removed from the house that prevented the health workers’ access to wall surfaces. This would modify some of the observed spray coverage rate values.

In this study, we adapted the recently developed IQK™ chemistry to estimate alpha-cypermethrin a.i. concentrations as described in Additional file 1. Previous studies have used the IQK™ chemistry to quantify pyrethroids, bendiocarb, and DDT delivered and residual concentrations on a variety of sampling mediums including adhesive tape, felt pads, glue dots, long-lasting insecticidal nets (LLINs), and in scrapings from treated wall surfaces [39, 47, 53–55]. Unlike the previous methods, here, we adapted the IQK™ assay conditions to quantify alpha-cypermethrin a.i. captured onto filter papers and in tank solution samples. We demonstrate that the values generated by the IQK™ were strongly correlated (*r*^2^ = 0.93) with those estimated using HPLC analysis, considered the gold-standard method. A similarly high correlation was reported between estimates using the IQK™ benchmarked against HPLC during evaluation of residual deltamethrin concentrations in LLINs [55]. Consequently, it is not likely that the observed variation in the insecticide concentrations in this study were due to inaccuracy in quantification methods. The efficiency of filter papers in capturing alpha-cypermethrin WP and water-dispersible granule (WG) formulations is high [56], and should be similar to the SC formulation assessed here. Data on the potential insecticide degradation during filter paper storage, or loss of a.i. by friction during transportation, would be useful. HPLC analysis is relatively expensive and requires specialist equipment and a high level of staff training, and thus is not usually suited to endemic settings. By contrast, the IQK™ is low-tech, providing readings within 30 min, and cost-effective (< $10 per assay), making it a useful tool to locally support quality control, training, and decision-making regarding equipment performance (e.g. [40, 53]).

WHO guidelines to measure insecticide delivery to houses are to locate at least four filter papers on different walls and at different wall heights prior to spraying [43]. The use of filter papers for insecticide capture is a logistically easy method, but being visible to spray teams, potentially could influence their performance. In this study, we noted that the spray coverage rates in houses without filter papers (15.4 m^2^/min) were significantly greater than in houses with filter papers fitted (9.23 m^2^/min). However, as only one of the three health workers were monitored in this sample, the suggested influence of filter paper presence on health worker behaviour should be treated with caution. Nonetheless, filter papers, or other a.i.-capturing materials such as small felt pads [39], could be designed to be less conspicuous.

For logistical reasons, this study was limited in the numbers of houses that could be monitored, and the insecticide concentrations were only measured at a single time point. Whilst the study community appears typical of the local region, assessment of IRS practices and insecticide residual activity amongst a large number of IRS-treated communities and health workers would be informative.

### Implications of Chagas disease control

The consequences of poor vector control are far-reaching. Bolivian Chaco communities register childhood infection prevalence of 20–25%, with annual force of infection rates of 0.021 and 0.046 [18–20]. Following Bolivian Ministry of Health (MoH) guidelines, treatment of chronically infected children (children under 15 years old, positive for *T. cruzi* antibodies) is advised only once communities are under successful vector control, defined as ≤ 3% of the houses in the community infested (the infestation rate), and proven absence of triatomine nymphal stages in the patient’s house [29]. Thus, confirmation of a successful vector control programme also relies on accurate triatomine surveillance methods, which currently is based on timed manual capture [57], but which is criticised for its low sensitivity [58], particularly to detect infestations in wall crevices [59]. Residual house infestation post-IRS is commonly observed in the Gran Chaco region [60–62]. The longer-term consequences of successive suboptimal exposure to insecticides can lead to genotype selection in the vector population promoting insecticide resistance or tolerance [63], as already reported in the Chaco region [22, 64].

## Conclusions

IRS is the only vector control activity against *Tri. infestans* transmission in the Chaco region. The collective results of this study demonstrate the generally known abiotic and biotic complexities in achieving effective and sustainable chemical control. The suboptimal a.i. concentrations observed here were partially attributable to the insecticide physical characteristics, but also indicate the need for revision of IRS practices, including training of health workers and community education to encourage compliance. The quality of IRS delivery and coverage will have an important bearing on the residual insecticidal effectiveness in houses, and thus on the interval between required IRS campaigns. Strategies to improve the rigour of IRS practices in the region are possible [65], and the IQK™ is an available tool to facilitate the much-needed changes.

## Supplementary Information

**Additional file 1.** Protocol of laboratory assays.

**Additional file 2.** Median alpha-cypermethrin a.i. concentrations on filter papers fitted to houses at different wall heights (*n *= 158 at 0.2 m; *n* = 157 at 1.2 m; *n* = 165 at 2 m). The horizontal lines represent the alpha-cypermethrin a.i. target concentration range (50 mg ± 20% a.i./m^2^). Error bars represents the lower and upper adjacent values to the median; points represent the outside values.

**Additional file 3.** Frequency distribution of the observed/expected spray rates in IRS-treated houses (*n* = 80 houses).

**Additional file 4.** Variation in the spray rates by individual health workers (A) median observed spray rates (m^2^/min); and (B) observed/expected spray rate ratio where the expected ratio is 19 m^2^/min for the spraying equipment calibration.

**Additional file 5.** (A) Median spray rates (m^2^/min) in IRS-treated houses with (+ F.P.) and without (− F.P.) filter papers fitted to the walls pre-spraying, stratified by the level of householder compliance with the request to empty their houses in preparation for IRS application (*n* = 55 houses); (B) Median insecticide a.i. concentrations (mg/m^2^) delivered to houses stratified by householder compliance (*n* = 25 houses).

## Data Availability

The data supporting the conclusions of this article are included within the article. Data are available from the corresponding authors on reasonable request to be used solely within the context of this study, following the ethical agreements, and with permission from the relevant authorities and co-authors.

## References

[1] Bern C (2015). Chagas' disease. N Engl Med.

[2] GBD 2017 DALYs and HALE Collaborators. Global, regional, and national disability-adjusted life-years (DALYs) for 359 diseases and injuries and healthy life expectancy (HALE) for 195 countries and territories, 1990–2017: a systematic analysis for the Global Burden of Disease Study 2017. Lancet. 2018; 392(10159):1859–1922. 10.1016/S0140-6736(18)32335-3 PMC6252083 30415748

[3] GBD 2017 Diseases and Injury Incidence and Prevalence Collaborators. Global, regional, and national incidence, prevalence, and years lived with disability for 354 diseases and injuries for 195 countries and territories, 1990–2017: a systematic analysis for the Global Burden of Disease Study. Lancet. 2018; 392(10159):1789–1858. 10.1016/S0140-6736(18)32279-7 PMC6227754 30496104

[4] GBD 2017 Causes of Death Collaborators. Global, regional, and national age- sex- specific mortality for 282 causes of death in 195 countries and territories, 1980–2017: a systematic analysis for the Global Burden of Disease Study 2017. Lancet. 2018; 392(10159):1736–1788. 10.1016/S0140-6736(18)32203-7 PMC6227606 30496103

[5] WHO. World Health Organization. Chagas disease in Latin America: an epidemiological update based on 2010 estimates. 2015. http://www.who.int/wer Accessed 29 May 2021. 25671846

[6] Pinazo MJ, Munoz J, Posada E, Lopez-Chejade P, Gallego M, Ayala E (2010). Tolerance of benznidazole in treatment of Chagas' disease in adults. Antimicrob Agents Chemother.

[7] Lee BY, Bacon KM, Bottazzi ME, Hotez PJ (2013). Global economic burden of Chagas disease: a computational simulation model. Lancet Infect Dis.

[8] Munoz J, Coll O, Juncosa T, Verges M, del Pino M, Fumado V (2009). Prevalence and vertical transmission of *Trypanosoma cruzi* infection among pregnant Latin American women attending 2 maternity clinics in Barcelona. Spain Clin Infect Dis.

[9] Liu Q, Zhou XN (2015). Preventing the transmission of American trypanosomiasis and its spread into non-endemic countries. Infect Dis Poverty.

[10] Schofield CJ, Jannin J, Salvatella R (2006). The future of Chagas disease control. Trends Parasitol.

[11] De Urioste-Stone SM, Pennington PM, Pellecer E, Aguilar TM, Samayoa G, Perdomo HD (2015). Development of a community-based intervention for the control of Chagas disease based on peridomestic animal management: an eco-bio-social perspective. Trans R Soc Trop Med Hyg.

[12] Dias JC, Silveira AC, Schofield CJ (2002). The impact of Chagas disease control in Latin America: a review. Mem Inst Oswaldo Cruz.

[13] Gurevitz JM, Ceballos LA, Sol Gaspe M, Alvarado-Otegui JA, Enriquez GF, Kitron U (2011). Factors affecting infestation by *Triatoma infestans* in a rural area of the Humid Chaco in Argentina: a multi-model inference approach. PLoS Negl Trop Dis..

[14] Gürtler RE, Yadon ZE (2015). Eco-bio-social research on community-based approaches for Chagas disease vector control in Latin America. Trans R Soc Trop Med Hyg.

[15] Dias JCP (2007). Southern Cone Initiative for the elimination of domestic populations of Triatoma infestans and the interruption of transfusional Chagas disease: historical aspects, present situation, and perspectives. Mem Inst Oswaldo Cruz..

[16] Dias JCP, Schofield CJ (1999). The evolution of Chagas disease (American trypanosomiasis) control after 90 years since Carlos Chagas discovery. Mem Inst Oswaldo Cruz.

[17] Ministerio de Hacienda. Secretaría de Hacienda. Dirección de Asuntos Provinciales. Chaco. Informe sintético de caracterización socio-productiva. 2018. http://www2.mecon.gov.ar/hacienda/dinrep/Informes/archivos/chaco.pdf. Accessed 29 May 2021.

[18] Chippaux J-P, Postigo JR, Santalla JA, Schneider D, Brutus L (2008). Epidemiological evaluation of Chagas disease in a rural area of southern Bolivia. Trans R Soc Trop Med Hyg.

[19] Samuels AM, Clark EH, Galdos-Cardenas G, Wiegand RE, Ferrufino L, Menacho S (2013). Epidemiology of and impact of insecticide spraying on Chagas disease in communities in the Bolivian Chaco. PLoS Negl Trop Dis..

[20] Hopkins T, Goncalves R, Mamani J, Courtenay O, Bern C (2019). Chagas disease in the Bolivian Chaco: persistent transmission indicated by childhood seroscreening study. Int J Infec Dis.

[21] Lardeux F, Depickere S, Aliaga C, Chavez T, Zambrana L (2015). Experimental control of *Triatoma infestans* in poor rural villages of Bolivia through community participation. Trans R Soc Trop Med Hyg.

[22] Santo-Orihuela PL, Vassena CV, Carvajal G, Clark E, Menacho S, Bozo R (2017). Toxicological, enzymatic, and molecular assessment of the insecticide susceptibility profile of *Triatoma infestans* (Hemiptera: Reduviidae, Triatominae) populations from rural communities of Santa Cruz. Bolivia J Med Entomol.

[23] Perez E, Monje M, Chang B, Buitrago R, Parrado R, Barnabe C (2013). Predominance of hybrid discrete typing units of *Trypanosoma cruzi* in domestic *Triatoma infestans* from the Bolivian Gran Chaco region. Infect Genet Evol.

[24] Wilson AL, Courtenay O, Kelly-Hope LA, Scott TW, Takken W, Torr SJ (2020). The importance of vector control for the control and elimination of vector-borne diseases. PLoS Negl Trop Dis..

[25] WHO. World Health Organization. Global vector control response 2017–2030.2017. https://www.paho.org/en/documents/global-vector-control-response-2017-2030 Accessed 29 May 2021.

[26] Dias JCP (1987). Control of Chagas disease in Brazil. Parasitol Today.

[27] Aiga H, Sasagawa E, Hashimoto K, Nakamura J, Zuniga C, Chevez JER (2012). Chagas disease: assessing the existence of a threshold for bug infestation rate. Am J Trop Med Hyg.

[28] OPAS. Organización Panamericana de la Salud. Taller para el establecimiento de pautas tecnicas en el control de *Triatoma dimidiata*. 2002. https://www.paho.org/uru/index.php?option=com_docman&view=download&alias=58-taller-para-el-establecimiento-de-pautas-tecnicas-en-el-control-de-tiatoma-dimidiata&category_slug=manuales-y-guias&Itemid=307. Accessed 29 May 2021.

[29] Bolivia. Ministerio de Salud y Deportes. Unidad de Epidemiologia, Programa Nacional de Chagas. Manual de procesos para la detección, diagnóstico, tratamiento y seguimiento de la enfermedad de Chagas infantil. 2007. https://www.minsalud.gob.bo/images/Documentacion/dgss/Epidemiologia/NORMATIVOS%20PNCH/Manual%20de%20Procesos%2031.pdf. Accessed 29 May 2021.

[30] Gurtler RE (2009). Sustainability of vector control strategies in the Gran Chaco Region: current challenges and possible approaches. Mem Inst Oswaldo Cruz.

[31] Gurtler RE, Kitron U, Cecere MC, Segura EL, Cohen JE (2007). Sustainable vector control and management of Chagas disease in the Gran Chaco, Argentina. Proc Natl Acad Sci USA.

[32] de Arias A, Lehane MJ, Schofield CJ, Fournet A (2003). Comparative evaluation of pyrethroid insecticide formulations against *Triatoma infestans* (Klug): residual efficacy on four substrates. Mem Inst Oswaldo Cruz..

[33] de Arias A, Lehane MJ, Schofield CJ, Maldonado M (2004). Pyrethroid insecticide evaluation on different house structures in a Chagas disease endemic area of the Paraguayan Chaco. Mem Inst Oswaldo Cruz..

[34] Picollo MI, Vassena C, Orihuela PS, Barrios S, Zaidemberg M, Zerba E (2005). High resistance to pyrethroid insecticides associated with ineffective field treatments in *Triatoma infestans* (Hemiptera : Reduviidae) from northern Argentina. J Med Entomol.

[35] Zerba EN (1999). Susceptibility and resistance to insecticides of Chagas disease vectors. Medicina-Buenos Aires.

[36] Guillen G, Diaz R, Jemio A, Cassab JA, Pinto CT, Schofield CJ (1997). Chagas disease vector control in Tupiza, southern Bolivia. Mem Inst Oswaldo Cruz.

[37] Coleman M, Foster GM, Deb R, Singh RP, Ismail HM, Shivam P (2015). DDT-based indoor residual spraying suboptimal for visceral leishmaniasis elimination in India. Proc Natl Acad Sci USA.

[38] Chowdhury R, Huda MM, Kumar V, Das P, Joshi AB, Banjara MR (2020). The Indian and Nepalese programmes of indoor residual spraying for the elimination of visceral leishmaniasis: performance and effectiveness. Ann Trop Med Parasitol..

[39] Russell TL, Morgan JC, Ismail H, Kaur H, Eggelte T, Oladepo F (2014). Evaluating the feasibility of using insecticide quantification kits (IQK) for estimating cyanopyrethroid levels for indoor residual spraying in Vanuatu. Malar J.

[40] Kaur H, Eggelte T: In Colorimetric assay for pyrethroid insecticides, Vol. WO/2009/106845, G01N 31/22 (2006.01) edition. Edited by World Intellectual Property Organization; 2009. - In Colorimetric assay. 2009.

[41] Pennington RT, Prado DE, Pendry CA (2000). Neotropical seasonally dry forests and Quaternary vegetation changes. J Biogeogr.

[42] WHO. World Health Organization. Manual para el rociado residual intradomiciliario - Aplicación del rociado para el control de vectores. 2002. https://www.paho.org/hq/dmdocuments/2014/Manual-para-el-rociado-residual-intradomicilario--2002.pdf Accessed 29 May 2021.

[43] WHO. World Health Organization. Guidelines for testing mosquito adulticides for indoor residual spraying treatment of mosquito nets. 2006. https://apps.who.int/iris/bitstream/handle/10665/69296/WHO_CDS_NTD_WHOPES_GCDPP_2006.3_eng.pdf?sequence=1&isAllowed=y. Accessed 29 May 2021.

[44] Rozendaal JA, World Health Organization. Vector control: methods for use by individuals and communities. 1997. https://www.who.int/whopes/resources/vector_rozendaal/en/ Accessed 29 May 2021.

[45] StataCorp: Stata Statistical Software: Release 15. College Station, TX: StataCorp LLC. https://www.stata.com/ (2017).

[46] Chowdhury R, Chowdhury V, Faria S, Islam S, Maheswary NP, Akhter S (2018). Indoor residual spraying for kala-azar vector control in Bangladesh: a continuing challenge. PLoS Negl Trop Dis..

[47] Ismail HM, Kumar V, Singh RP, Williams C, Shivam P, Ghosh A (2016). Development of a simple dipstick assay for operational monitoring of DDT. PLoS Negl Trop Dis..

[48] Tan C-x, Shen D-l, Weng J-q, Chen Q-w, Liu H-j, Yuan Q-l (2004). Research on the rheological properties of pesticide suspension concentrate. J Zhejiang Univ Sci.

[49] Gorla D, Hashimoto K, Telleria J, Tibayrenc M (2017). Control strategies against Triatominae. American Trypanosomiasis Chagas Disease.

[50] Huda MM, Mondal D, Kumar V, Das P, Sharma SN, Das ML (2011). Toolkit for monitoring and evaluation of indoor residual spraying for visceral leishmaniasis control in the Indian subcontinent: application and results. J Trop Med..

[51] Fuseini G, Ismail HM, von Fricken ME, Weppelmann TA, Smith J, Logan RAE (2020). Improving the performance of spray operators through monitoring and evaluation of insecticide concentrations of pirimiphos-methyl during indoor residual spraying for malaria control on Bioko Island. Malar J.

[52] Paz-Soldan VA, Bauer KM, Hunter GC, Castillo-Neyra R, Arriola VD, Rivera-Lanas D (2018). To spray or not to spray? Understanding participation in an indoor residual spray campaign in Arequipa. Peru Glob Public Health.

[53] Kumar V, Mandal R, Das S, Kesari S, Dinesh DS, Pandey K (2020). Kala-azar elimination in a highly-endemic district of Bihar, India: A success story. PLoS Negl Trop Dis..

[54] Thawer NG, Ngondi JM, Mugalura FE, Emmanuel I, Mwalimu CD, Morou E (2015). Use of insecticide quantification kits to investigate the quality of spraying and decay rate of bendiocarb on different wall surfaces in Kagera region. Tanzania Parasit Vectors.

[55] Kaur H, Allan EL, Eggelte TA, Monti F. A Colorimetric test for the evaluation of the insecticide content of LLINs used on Bioko Island, Equatorial Guine. Preprint at https://assets.researchsquare.com/files/rs-64354/v1/e2d6eccc-919a-4a30-9293-5cd027fb9dad.pdf. (2020). 10.1186/s12936-021-03967-w PMC8579635 34758840

[56] Moiroux N, Djenontin A, Zogo B, Bouraima A, Sidick I, Pigeon O (2018). Small-scale field testing of alpha-cypermethrin water-dispersible granules in comparison with the recommended wettable powder formulation for indoor residual spraying against malaria vectors in Benin. Parasit Vectors.

[57] Schofield CJ (1978). A comparison of sampling techniques for domestic populations of Triatominae. Trans R Soc Trop Med Hyg.

[58] Abad-Franch F, Valenca-Barbosa C, Sarquis O, Lima MM (2014). All that glisters is not gold: sampling-process uncertainty in disease-vector surveys with false-negative and false-positive detections. PLoS Negl Trop Dis..

[59] Rabinovich JE, Gurtler RE, Leal JA, Feliciangeli D (1995). Density estimate of the domestic vector of Chagas disease, *Rhodnius prolixus* Stål (Hemiptera:Reduviidae) in rural houses in Venezuela. Bull World Health Organ.

[60] Provecho YM, Gaspe MS, Fernandez MD, Gurtler RE (2017). House reinfestation with *Triatoma infestans* (Hemiptera: Reduviidae) after community-wide spraying with insecticides in the Argentine Chaco: a multifactorial process. J Med Entomol.

[61] Cecere MC, Rodriguez-Planes LI, Vazquez-Prokopec GM, Kitron U, Gurtler RE (2019). Community-based surveillance and control of chagas disease vectors in remote rural areas of the Argentine Chaco: A five-year follow-up. Acta Trop.

[62] Gurevitz JM, Sol Gaspe M, Enriquez GF, Provecho YM, Kitron U, Guertler RE (2013). Intensified surveillance and insecticide-based control of the Chagas Disease vector *Triatoma infestans* in the Argentinean Chaco. PLoS Negl Trop Dis..

[63] Mougabure-Cueto G, Picollo MI (2015). Insecticide resistance in vector Chagas disease: Evolution, mechanisms and management. Acta Trop.

[64] Roca-Acevedo G, Ines Picollo M, Santo-Orihuela P (2013). Expression of insecticide resistance in immature life stages of *Triatoma infestans* (Hemiptera: Reduviidae). J Med Entomol.

[65] Carla Cecere M, Vazquez-Prokopec GM, Ceballos LA, Boragno S, Zarate JE, Kitron U (2013). Improved chemical control of Chagas Disease vectors in the Dry Chaco region. J Med Entomol.

