# Supplementary material for: Indoor residual spraying practices against Triatoma infestans in the Bolivian Chaco: contributing factors to suboptimal insecticide delivery to treated households
Source: Parasit Vectors. 2021 Jun 16;14:327. doi: 10.1186/s13071-021-04831-1 (PMC8207695; doi:10.1186/s13071-021-04831-1)
Supplement: Supplementary file 4 — Additional file 4. Variation in the spray rates by individual health workers (A) median observed spray rates (m2/min); and (B) observed/expected spray rate ratio where the expected ratio is 19 m2/min for the spraying equipment calibration. [file 13071_2021_4831_MOESM4_ESM.docx]

(A)


(B)
