# Supplementary material for: Indoor residual spraying practices against Triatoma infestans in the Bolivian Chaco: contributing factors to suboptimal insecticide delivery to treated households
Source: Parasit Vectors. 2021 Jun 16;14:327. doi: 10.1186/s13071-021-04831-1 (PMC8207695; doi:10.1186/s13071-021-04831-1)
Supplement: Supplementary file 3 [file 13071_2021_4831_MOESM2_ESM.docx]

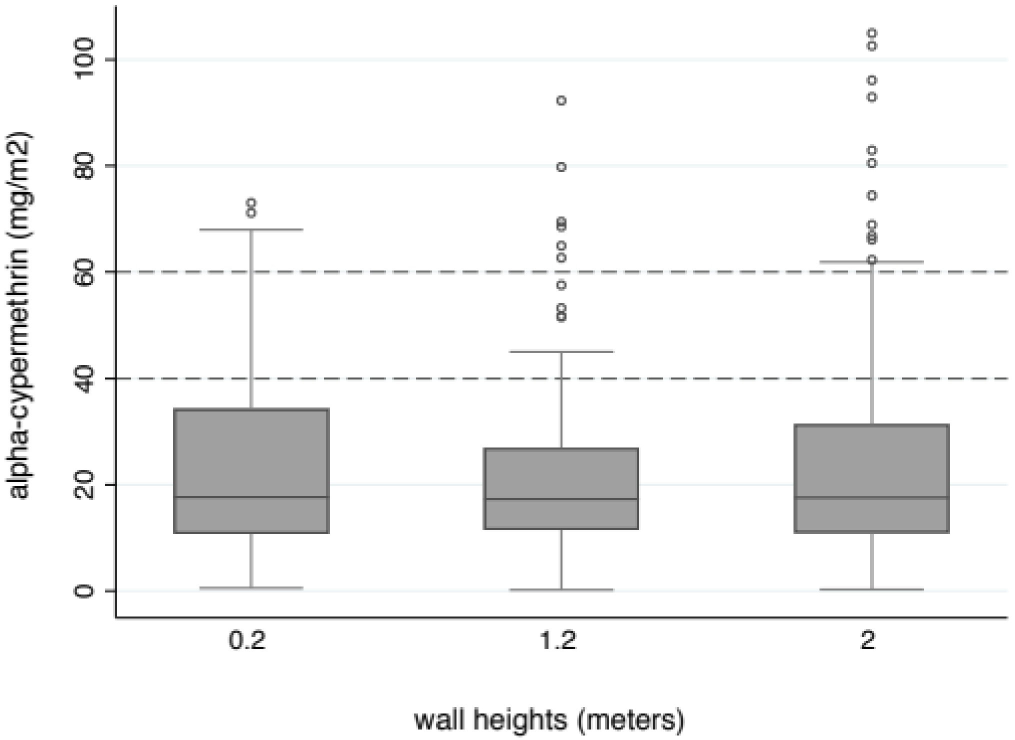

alpha-cypermethrin (mg/m2)
0
20
40
60
80
100
0.2
1.2
2
wall heights (meters)
